# Cocaine to prevent bleeding during nasotracheal intubation: A systematic review

**DOI:** 10.1111/aas.70002

**Published:** 2025-02-16

**Authors:** Mo Haslund Larsen, Oscar Rosenkrantz, Mette Krag, Lars Simon Rasmussen, Dan Isbye

**Affiliations:** ^1^ Department of Anaesthesia Centre of Head and Orthopaedics, Copenhagen University Hospital Copenhagen Denmark; ^2^ Department of Clinical Epidemiology Aarhus University & Aarhus University Hospital Aarhus Denmark; ^3^ Danish Ministry of Defence, Personnel Agency Copenhagen Denmark; ^4^ Emergency Medical Services Capital Region of Denmark Denmark

**Keywords:** cocaine, epistaxis, nasotracheal intubation

## Abstract

**Background:**

Nasotracheal intubation is associated with a risk of epistaxis. Decongestion of the nasal mucosa reduces the risk of epistaxis, and different vasoconstrictors may be used. Cocaine has both decongestive and analgesic properties, but it also has side effects. In this systematic review, we aimed to evaluate if cocaine decreases the occurrence and severity of epistaxis when administered topically to the nasal mucosa before nasotracheal intubation.

**Methods:**

We conducted a systematic review and meta‐analysis following the PRISMA guidelines based on a predefined protocol. We included randomized clinical trials comparing nasal cocaine to active comparators or placebo for nasotracheal intubation. Two reviewers independently screened studies for eligibility and performed data extraction. Relative risk with 95% confidence intervals was calculated. Predefined primary outcome measures were the occurrence and severity of epistaxis. Secondary outcomes were pain, mechanical complications, and patient‐centered side effects. The risk of bias was evaluated using the revised Cochrane Risk of Bias 2 tool for randomized trials, and certainty of evidence on outcome level was assessed according to GRADE.

**Results:**

Six trials (*n* = 457) were included; one trial was judged as having a low risk of bias. All six trials provided information on the occurrence of epistaxis. The meta‐analysis did not support a difference in the occurrence of epistaxis between cocaine and its comparators (fixed effect: relative risk 0.90 [95% confidence interval 0.75 to 1.09, *I* ^2^ of 0%, certainty of evidence: low]). The severity of epistaxis was evaluated on incompatible scales and thus not suitable for meta‐analysis. No studies reported on pain or mechanical complications associated with nasotracheal intubation, and data on patient‐centered side effects were sparse.

**Conclusion:**

This systematic review with meta‐analysis demonstrated that the quantity and certainty of evidence on cocaine used for nasotracheal intubation is low and that there is no firm evidence for the benefits and harms of cocaine compared to other vasoconstrictors and topical analgetics or placebo. Consequently, sufficiently powered randomized trials assessing patient‐centered outcomes, including outcomes on side effects, should be conducted before firm conclusions on cocaine for nasotracheal intubation can be drawn.

**Editorial Comment:**

Epistaxis can occur with nasotracheal intubation, and topical drug vasoconstrictor effects have been used to reduce this risk. This analysis shows that the evidence base supporting the use of cocaine for reducing the risk of epistaxis in nasotracheal intubation is uncertain.

## 1 INTRODUCTION

Nasotracheal intubation is used in patients where oral intubation is not a viable option.^1^ Decongestion of the nasal mucosa is used to reduce the risk of epistaxis during the procedure, and in patients undergoing awake fiberoptic nasotracheal intubation, decongestion is complemented by topical analgesia.^2^ The incidence of epistaxis in relation to nasotracheal intubation varies between 24% and 93%, depending on definitions, techniques, and devices used.^3^

Cocaine has been used for nasal preparation before nasotracheal intubation due to its unique combination of both decongestive and analgesic properties.^4^, ^5^ Vasoconstriction arises from cocaine's inhibition of reuptake and metabolism of catecholamines and analgesia occurs through blockage of neuronal sodium channels, thus inhibiting propagation of action potentials.^6^ Despite the benefits of cocaine's dual action, rising concerns of patient‐centered side effects, cardiac adversity and toxicity have increased the interest for worthy alternatives.^7^, ^8^, ^9^, ^10^ Several other vasoconstrictive and analgetic agents have been assessed, but it remains unclear if these alternatives are superior to cocaine. Currently, no widely accepted guidelines exist on nasal preparation before nasotracheal intubation.^11^ Hence, this systematic review aimed to assess the efficacy of cocaine in decreasing the occurrence and severity of epistaxis when administered topically to the nasal mucosa before nasotracheal intubation compared to other medical interventions or placebo. We also wished to explore the effect on pain, the occurrence of mechanical complications to the procedure, and patient‐centered side effects.

## 2 METHODS

The manuscript for this systematic review was prepared according to the Preferred Reporting Items for Systematic Reviews and Meta‐Analysis (PRISMA) statement,^12^ followed the Cochrane Handbook for Systematic Reviews of Interventions^13^ and used the GRADE tool^14^ to evaluate the certainty of the evidence of included trials. Before the commencement of screening, the protocol was registered in the International Prospective Register of Systematic Reviews (PROSPERO, no. CRD42023428600).

### 2.1 Eligibility criteria

We planned to include interventional trials (randomized and non‐randomized) and observational studies assessing adult, elective surgical patients, or healthy volunteers. Studies were included regardless of publication year, publication status, blinding, and language. In the protocol, adults were defined as participants at 18 years or older, but coming across two trials defining adults as participants at 16 years or older, we decided to include these trials in this review. The intervention of interest was cocaine administered to the nasal mucosa, and the comparators were other topical vasoconstrictors and analgesic agents including, but not limited to, epinephrine, oxymetazoline/xylometazoline, phenylephrine, lidocaine, and placebo. We excluded trials in animals and studies on cocaine as an intoxicant.

### 2.2 Outcome measures

The primary outcome measures were the occurrence and severity of epistaxis immediately following nasotracheal intubation as defined by the authors of the included studies. Secondary outcomes were pain, mechanical complications, and patient‐centered side effects.

### 2.3 Information sources and search strategy

We searched Medline, Embase, and the Cochrane Library on February 22nd, 2023. The search strategy was developed with a health information specialist and refined before the final search. A detailed search strategy is presented in Appendix S1. The latest search was conducted on August 1, 2024. We also searched the trial registries Clinicaltrials.gov and the World Health Organization's International Clinical Trials Registry Platform for unpublished and ongoing studies and hand‐searched reference lists of relevant trials and systematic reviews. The PROSPERO database was accessed on August 1, 2024, to check for other similar ongoing studies.

### 2.4 Selection process

Screening was conducted within the Covidence systematic review software (Veritas Health Innovation, Melbourne, Australia. Available at www.covidence.org) and duplicates were removed before initiating the screening process, first in the reference managing software Mendeley (Elsevier, Amsterdam, Netherlands. Available at www.mendeley.com) and then within Covidence. Two authors, M.H.L. and O.R., independently screened titles and abstracts from the primary search and excluded apparently irrelevant trials. Any discrepancies were resolved by discussion until an agreement was reached. The remaining studies were evaluated in full text. If only an abstract was found, the authors were contacted to obtain the full‐text version. The abstracts were excluded in the case of no response. When agreement could not be reached, a third author advised. Interrater reliability was calculated using Cohen's kappa statistic.^15^

### 2.5 Data extraction

Two authors, M.H.L. and O.R., independently extracted data using a standardized data extraction template. The data extracted consisted of trial characteristics, patient characteristics, data on intervention and comparator and outcome measures. Missing information was classified as either not available or not applicable.

### 2.6 Risk of bias assessment

Two authors, M.H.L. and O.R., independently evaluated the risk of bias on outcome level regarding epistaxis using the revised Cochrane Risk of Bias 2 tool for randomized trials.^16^ Risk of bias levels was categorized as “low risk,” “some concerns,” or “high risk” based on the domain randomization process, deviations from the intended interventions, missing outcome data, measurement of the outcome, and selection of the reported result. A third author resolved discrepancies in the risk of bias evaluation. No automation tools were used.

### 2.7 GRADE

The certainty of evidence on outcome level was assessed according to GRADE.^14^ In this method, the certainty of the evidence for the outcome is rated “very low,” “low,” “moderate” or “high”. Randomized clinical trials begin with a high certainty of evidence, while observational studies begin with a low certainty of evidence. Certainty of evidence can be rated down for risk of bias, imprecision, inconsistency, indirectness, and likelihood of publication bias.

### 2.8 Data synthesis

We used the systematic review software Review Manager (RevMan, version 5.4, The Cochrane Collaboration, 2020). For each included trial, we calculated the relative risk (RR) with a 95% confidence interval (CI) for dichotomous outcome measures using the number of participants and events per group.

We quantified statistical heterogeneity between trials with inconsistency factor (*I* ^2^) and diversity (*D* ^2^) statistics.^17^ If *I* ^2^ was 0, we used and reported results from a fixed‐effect model, and if *I* ^2^ > 0, we used and reported results from a random‐effects model.

## 3 RESULTS

**FIGURE 1:**
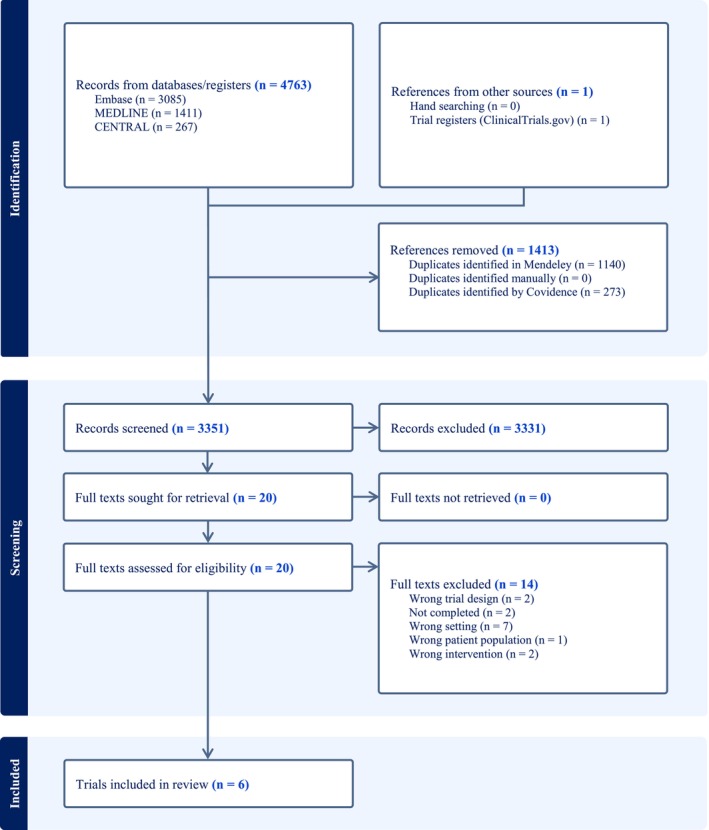
Identification of studies assessing cocaine for nasotracheal intubation.

After removing 1413 duplicates, 3351 records underwent title and abstract screening. Of these, 3331 studies were excluded, leaving 20 for full‐text screening, with full‐text articles available for all 20 records. Six studies with a total of 457 patients were included in this review^18^, ^19^, ^20^, ^21^, ^22^, ^23^ (Figure 1). The kappa coefficient was 0.89 for title and abstract screening and 0.76 for full‐text screening, indicating almost perfect agreement and substantial agreement, respectively. All the included studies were randomized clinical trials. The main reason for the exclusion of trials was the wrong setting (not nasotracheal intubation).

### 3.1 Trial characteristics

All trials were published in English, and all were single‐center trials. Three trials were conducted in the United States,^18^, ^19^, ^23^ two in Europe^20^, ^21^ and one in Asia.^22^ All six trials were included in the meta‐analysis of the primary outcome. Details are provided in Table 1.

**TABLE 1:** Characteristics of the included trials.

Author	No. of patients^a^	Setting	Clinical setting	Inclusion criteria	Intervention (cocaine)	Comparator(s)	Method of administration	Outcomes
Gross^18^	99	United States	Oral surgery outpatients	ASA 1, nothing by mouth, minimum 8 h, no medications before start of trial	0.5 mL 4%	0.5 mL Phenylephrine 0.25% + Lidocaine 3% 0.5 mL Phenylephrine 0.25%	Multiorificed cannula	Epistaxis
Katz^19^	42	United States	Oral surgery patients	≥16 years, nasotracheal intubation for oral surgery	2 mL 10%	2 mL Lidocaine 4% + Epinephrine 0.001% 2 mL oxymetazoline 0.05%	Saturated cotton swabs were placed in nares for 5 min	Epistaxis
Larsen^20^	102	Denmark	Maxillofacial surgery patients	≥18 years, nasotracheal intubation for maxillofacial surgery	2 mL 4%	2 mL Xylometazoline 0.05%	Intranasal mucosa atomization device	Epistaxis, Side effects
Latorre^21^	99	Germany	Maxillo‐facial surgery patients	Nasotracheal intubation for maxillofacial surgery	0.5 mL 10%	0.5 mL Phenylephrine 0.25% + Lidocaine 3%	Not reported	Epistaxis
Lu^22^	79	Taiwan	Oromaxillo‐facial surgery patients	Not reported	1 mL 4%	1 mL cocaine 6%	Intranasal mucosa atomization device	Epistaxis
Rector^23^	36	United States	Patients undergoing molar extraction	Young adults, healthy ASA I, general nasotracheal anesthesia	0.5 mL 10%	1 mL 0.1% Oxymetazoline 0.5 mL saline	Administered as a spray (Modified Devilbiss model 15 atomizer)	Epistaxis

*Note*: All were randomized trials assessing cocaine for nasotracheal intubation.

^a^ Studies with two randomization arms randomized 1:1 and studies with three randomization arms randomized 1:1:1.

### 3.2 Description of interventions

The dose of cocaine varied across studies from 0.5 mL 4% cocaine to 2 mL 10% cocaine. In four of the six studies, administration was through some form of atomizer, converting a liquid to a fine spray.^18^, ^20^, ^22^, ^23^ In five of the six studies, the comparators were alfa‐adrenergic agonists with phenylephrine with or without lidocaine or oxymetazoline/xylometazoline.^18^, ^19^, ^20^, ^21^, ^23^ Half the studies had three intervention arms,^18^, ^19^, ^23^ with one trial, including a placebo arm.^23^ The other half had two intervention arms, none of which were placebo.^20^, ^21^, ^24^ Of the 457 included patients, 207 received cocaine, and 250 received a comparator, of which 12 patients received a placebo. Details on interventions and comparators are available in Table 1.

### 3.3 Outcomes

#### 3.3.1 Epistaxis occurrence

**FIGURE 2:**
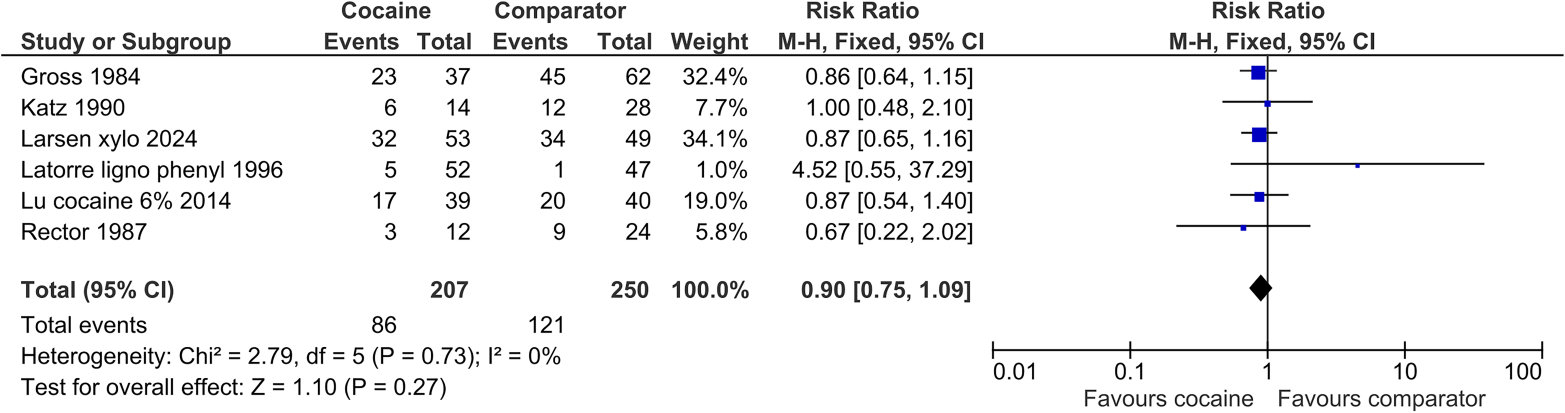
Forest plot of occurrence of epistaxis in trials comparing cocaine with comparators for nasotracheal intubation. The size of squares for relative risk (RR) reflects the weight of the trial in pooled analyses. Horizontal bars represent a 95% confidence interval (CI).

All six trials provided information on the occurrence of epistaxis. The conventional meta‐analysis showed no difference in the occurrence of epistaxis between cocaine and its comparators (457 patients, fixed effect: RR 0.90 [95% CI 0.75 to 1.09, *I* ^2^ of 0%]) (Figure 2).

A meta‐analysis excluding patients in the placebo arm did not change the conclusion (fixed effect: RR 0.92 [95% CI 0.76 to 1.11, *I* ^2^ of 0%]) (Appendix S2).

### 3.4 Risk of bias

**FIGURE 3:**
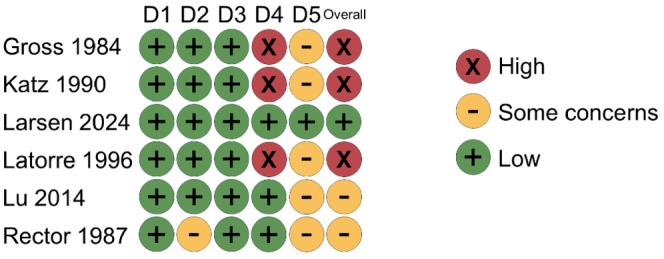
Risk of bias for the outcome of epistaxis using Cochrane's RoB2 tool on trials assessing cocaine for nasotracheal intubation. Domains: D1: Bias due to randomization. D2: Bias due to deviations from the intended intervention. D3: Bias due to missing data. D4: Bias due to outcome measurement. D5: Bias due to selection of reported result.

The risk of bias assessment for the outcome of the occurrence of epistaxis is depicted in Figure 3. One trial was judged as having a low risk of bias. Three trials were considered to have a high risk of bias, all resulting from a high risk of bias due to issues with outcome measurement.^18^, ^19^, ^21^ Two trials had some concerns about the risk of bias, with one arising from concerns with the selection of reported results,^22^ and the other selection of reported results and deviations from the intended intervention.^23^

### 3.5 GRADE assessment

The certainty of evidence for the outcome of epistaxis was low, downgraded for indirectness and imprecision (Table 2). As a result, no firm evidence for the prevention of epistaxis after nasotracheal intubation due to the administration of cocaine is available.

**TABLE 2:** GRADE assessment of the certainty of evidence for the outcome of epistaxis in trials assessing cocaine for nasotracheal intubation.^18^, ^19^, ^20^, ^21^, ^22^, ^23^

	Certainty assessment					
No. of trials	Risk of bias	Inconsistency	Indirectness	Imprecision	Other considerations	Certainty
Epistaxis						
6	Not serious^a^	None^b^	Serious^c^	Serious^d^	None^e^	⊕⊕◯◯

*Note*: Question: Efficacy of cocaine versus comparators or placebo before nasotracheal intubation to reduce the risk of epistaxis.

^a^ Results of one of three studies with high risk of bias comes to a different conclusion than all other studies.

^b^ General overlap of confidence intervals and comparable point estimates in forest plots and an *I* ^2^ of 0%.

^c^ Varying controls. Rated down by one level.

^d^ All studies were underpowered and had generally wide confidence intervals. Rated down by one level.

^e^ <10 studies so testing for publication bias was not conducted.

#### 3.5.1 Epistaxis severity

The degree of epistaxis was evaluated on numerical scales ranging from 2 to 5 points, but all had a choice of no bleeding/no epistaxis at one end of the scale. Different scales in the studies had varying criteria for the highest score. On one scale, the highest score was “frank epistaxis with blood in nose or mouth,”^18^ while on another scale, it was “bleeding complicating intubation.”^20^ Hence, the scales were not compatible and thus not suitable for meta‐analysis. A total of 52 out of this review's 457 patients received the highest score in their respective studies. Of these, 42 patients were from the trial, defining their most extreme degree of bleeding as “frank epistaxis with blood in nose or mouth.” See Appendix S3 for the complete bleeding scales.

### 3.6 Secondary outcomes

#### 3.6.1 Pain and mechanical complications

No studies reported pain or mechanical complications associated with nasotracheal intubation.

#### 3.6.2 Patient‐centered side effects

Patient‐centered side effects were only described in one trial.^20^ They reported headache after 24 h in 19% of the cocaine group and 29% of the comparator group (xylometazoline), and exaltation 5 min after drug administration in 15% of the cocaine group and 6% of the comparator group (xylometazoline). The trial found weak evidence of a difference between groups for headache and exaltation. No cases of arrhythmias or hyperthermia were observed. A risk of bias evaluation was not conducted as only one trial reported side effects.

## 4 DISCUSSION

In this systematic review of cocaine versus other medical interventions or placebo administered topically to the nasal mucosa, we found little to no difference in the occurrence of epistaxis during nasotracheal intubation. There were no data on pain or mechanical complications and too little data on patient‐centered side effects to draw conclusions.

Epistaxis is the most common complication during nasotracheal intubation^1^, ^24^, ^25^, ^26^ and massive epistaxis has life‐threatening potential.^27^, ^28^ When comparing different medical interventions for preparation of the nasal mucosa, the occurrence of epistaxis during the procedure is often used as an outcome measure as the absence of bleeding may indicate the potency of the drug's vasoconstrictive effect. However, lesser amounts of blood may not always be challenging or necessarily influence the safety of the procedure. Hence, the severity of bleeding may be a more clinically relevant outcome, but our study shows that this comes with other challenges. Of the 52 patients receiving the highest bleeding score on the scale used in their respective studies, 42 patients were from a trial that defined maximum bleeding as “frank epistaxis in the nose or mouth.”^18^ “Frank epistaxis” encompasses a wide range of severities and does not necessarily describe the bleeding's influence on the intubation or the safety of the patient. The remaining 10 high‐scoring patients came from trials that used a definition for their highest epistaxis score of greater clinical relevance, as these could compromise the intubation process. The significant differences in scoring scales combined with the small sample sizes make it difficult to draw firm conclusions on whether alternatives to cocaine are equally efficient in reducing clinically relevant bleeding.

The analgesic effect of cocaine compared to its alternatives for awake nasotracheal intubation is not well described, and none of the included studies addressed pain in relation to the procedure, as none of the studies included patients undergoing awake nasal fiberoptic intubation. One trial compared nasal administration of cocaine and phenylephrine/lidocaine in 25 healthy adults who had a nasotracheal tube inserted into the nasopharynx.^29^ The trial found no difference between the drugs in terms of their effect on pain.

Mechanical complications in relation to nasotracheal intubation occur during manipulation in a narrow cavity, and the degree of decongestion could affect the incidence and severity. Mechanical complications were not reported in any of the trials included in this review, even though it may be a clinically relevant outcome. Previous case reports have described mechanical complications of retropharyngeal dissection, dislocation or tear of a nasal turbinate, and nasal necrosis.^30^, ^31^, ^32^, ^33^ In an observational study, mainly including patients nasotracheally intubated in the emergency department, all nasotracheal intubation complications were recorded over two months, and retropharyngeal laceration was found in 2 of 71 intubations.^24^ However, there is a lack of data on mechanical complications related to nasotracheal procedures. Hence, we are unable to conclude whether one drug is superior to another in preventing it.

In the included studies, data on side effects were very sparse. One trial reported on headache and exaltation without differences between cocaine and the comparator.^20^ One trial reported on electrocardiogram changes and found no difference between groups.^21^ Three trials reported differences in heart rate and blood pressure, but using surrogate outcomes challenges clinically meaningful interpretations.^19^, ^20^, ^21^

The lack of reported side effects is interesting since case reports have contributed to concerns about the side effects of cocaine, especially in patients with cardiac disease.^8^ No causal relationship has been established, but incidents of takotsubo cardiomyopathy,^34^ myocardial ischemia^35^ and myocardial infarction^36^ following the administration of cocaine before nasal surgery have been published. A review described myocardial infarction arising in relation to either recreational or topical cocaine.^37^ They identified 114 cases of myocardial infarction attributed to cocaine. Of these, 35 had normal coronary arteries proven by angiography or autopsy, and the authors concluded that the causes of cardiac ischemia attributed to cocaine are complex and may be the result of combined coronary artery vasoconstriction, intracoronary thrombosis and atherosclerosis. Whether this association is present only in relation to the abuse of cocaine or can occur after a single administration is unknown. On the other hand, the association between cocaine and cardiotoxicity has been questioned. A study including 10,549 adult patients with no heart disease within 10 years of surgery found no significant increase in the risk of major perioperative cardiac events or mortality in patients receiving cocaine.^38^

The benefits and harms of cocaine administered before nasotracheal intubation remain debated. In this systematic review, data did not support a difference between cocaine and its comparators on the incidence of epistaxis. However, this conclusion should be interpreted with care as the quantity and certainty of evidence was low, and data describing the analgetic effects and side effects were lacking. Until firm data can guide clinicians, the choice of vasoconstrictor for nasotracheal intubation remains at the clinician's discretion, considering individual patient circumstances and clinical judgment.

### 4.1 Strengths and limitations

The strengths of our systematic review include a systematic search developed in collaboration with a health information specialist, the literature search conducted in three major databases Medline, Embase, and the Cochrane Library, a protocol registered in the PROSPERO database before commencement and adherence to the PRISMA guidelines for systematic reviews.^12^

Our review also has limitations. First, only one trial had a low risk of bias, which increases the risk of the overall findings being influenced by bias. Second, we did not foresee that studies used different bleeding scales and did not define a way to compare the severity of bleeding between studies. Hence, it was not possible to pool these data. Third, data on secondary outcomes were sparse, which challenges the interpretation and the ability to balance the benefits and harms of the different agents used. Fourth, we included two studies defining adults as ≥16 years, although it was protocolized to include participants ≥18 years. However, the important distinction intended was the separation of children and adults rather than a specific age, and we do not believe it introduces important biases. Fifth, variation in population and intervention of interest may give rise to heterogeneity. Finally, we included both studies comparing cocaine to placebo and active agents. Some may argue that pooling active agents and placebo in the same group and comparing them to cocaine may dilute the effect in the comparator group. However, very few patients were allocated to placebo, and the additional meta‐analysis excluding these patients did not change the conclusion on the occurrence of epistaxis.

### 4.2 Conclusion

This systematic review with meta‐analysis demonstrated that the quantity and certainty of the evidence on the efficacy of cocaine compared to other medical interventions or placebo in the prevention of epistaxis during nasotracheal intubation is low and that there is no firm evidence for the benefits and harms of cocaine compared to other vasoconstrictors and topical analgesics or placebo. Consequently, sufficiently powered randomized trials assessing patient‐centered outcomes, including outcomes on side effects, should be conducted before firm conclusions on cocaine for nasotracheal intubation can be drawn.

## AUTHOR CONTRIBUTIONS

The idea for this project was conceived by Lars Simon Rasmussen and Dan Isbye. Mo Haslund Larsen and Oscar Rosenkrantz screened records and performed data extraction. Mette Krag guided analysis and discussion. Mo H. Larsen wrote the first draft of this manuscript, which was critically revised and approved by all authors.

## FUNDING INFORMATION

The authors received no specific funding for this manuscript.

## CONFLICT OF INTEREST STATEMENT

MHL, OR, LSR, and DI authored a paper that is included in this review.

## Supporting information


**Data S1.** Supporting information.

## Data Availability

The data that support the findings of this study are available on request from the corresponding author. The data are not publicly available due to privacy or ethical restrictions.
