# Supplementary material for: Cocaine to prevent bleeding during nasotracheal intubation: A systematic review
Source: Acta Anaesthesiol Scand. 2025 Feb 16;69(3):e70002. doi: 10.1111/aas.70002 (PMC11830855; doi:10.1111/aas.70002)
Supplement: Supplementary file 1 — Data S1. Supporting information. [file AAS-69-0-s001.docx]

# Appendix 1: Search strategy

Ovid MEDLINE(R) ALL <1946 to January 17, 2024>

1 Administration, Intranasal/

2 vasoconstrictor agents/ or nasal decongestants/

3 (intranasal or intra-nasal or nasal or nose* or nasotracheal or topical* or detumescence or vasoconstrictor* or decongestant*).mp.

4 Nose/de, pd [Drug Effects, Pharmacology]

5 1 or 2 or 3 or 4

6 Cocaine/

7 cocain*.mp.

8 6 or 7

9 5 and 8

10 exp animals/ not humans.sh.

11 9 not 10

Ovid Embase <1974 to 2024 January 17>

1 intranasal drug administration/

2 vasoconstrictor agent/na, tp [Intranasal Drug Administration, Topical Drug Administration]

3 (intranasal or intra-nasal or nasal or nose* or nasotracheal or topical* or detumescence or vasoconstrictor* or decongestant*).mp.

4 nose/ or nose cavity/ or nose mucosa/

5 1 or 2 or 3 or 4

6 cocaine/

7 cocain*.mp.

8 6 or 7

9 5 and 8

10 (Animal experiment/ or animal/) not (human experiment/ or human/)

11 9 not 10

Cochrane Central Register of Controlled Trials

Issue 1 of 12, January 2024

#1 MeSH descriptor: [Administration, Intranasal] explode all trees

#2 MeSH descriptor: [Vasoconstrictor Agents] explode all trees

#3 MeSH descriptor: [Nasal Decongestants] explode all trees

#4 MeSH descriptor: [Nose] explode all trees and with qualifier(s): [drug effects - DE]

#5 (intranasal or intra-nasal or nasal or nose* or nasotracheal or topical* or detumescence or vasoconstrictor* or decongestant*):ti,ab,kw

#6 #1 or #2 or #3 or #4 or #5

#7 MeSH descriptor: [Cocaine] explode all trees

#8 cocain*:ti,ab,kw

#9 #7 or #8

#10 #6 and #9 in Trials

# Appendix 2: Meta-analysis not including patients allocated to placebo

**Supplemental figure 2:** Forest plot of occurrence of epistaxis in trials comparing cocaine versus comparators for nasotracheal intubation excluding patients allocated to placebo.


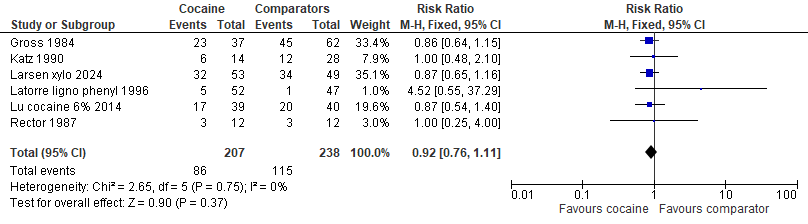


Size of squares for relative risk (RR) reflects the weight of the trial in pooled analyses, horizontal bars 95% confidence interval (CI).

All trials provided information on epistaxis. The conventional meta-analysis excluding 12 patients allocated to placebo showed no difference in the occurrence of epistaxis between cocaine and its comparators (fixed effect: RR 0.92 (95% CI 0.76 to 1.11, *I*^2^ of 0%).

# Appendix 3: Bleeding scales

| **Author** | **Bleeding scales** | | |
| --- | --- | --- | --- |
| Gross, 1984^18^ | **3-point scale evaluated visually at extubation**  0: No blood seen  1: Traces of blood on endotracheal tube  2: Frank epistaxis with blood in nose or mouth | | |
| Katz, 1990^19^ | **4-point scale evaluated visually by laryngoscopy at intubation**  0: No bleeding  1: Blood only on endotracheal tube  2: Blood in pharynx  3: Blood in pharynx sufficient to impede intubation | | |
| Larsen, 2024^20^ | **4-point scale evaluated visually by laryngoscopy at intubation**  0: No bleeding  1: Blood only on endotracheal tube  2: Blood in pharynx  3: Blood in pharynx sufficient to impede intubation | | |
| Latorre, 1996^21^ | **4-point scale evaluated by endoscopy at intubation**  0: No epistaxis  1: Epistaxis during endoscopy  2: Epistaxis during insertion of the nasotracheal tube  3: Profuse epistaxis confirmed by laryngoscopy | | |
| Lu, 2014^22^ | Visually evaluated at undisclosed times:  **Degree oropharyngeal space: 5-point scale**  0: None  1: Minimal, blood-tinged on oropharynx, but nasotracheal tube not infiltrated with blood  2: Mild, the tube infiltrated with blood with less than half the height immersed  3: Moderate, the tube infiltrated with blood and greater than half the height immersed  4: Severe, part of the tube totally immersed and invisible because of infiltration by blood | | |
| Rector, 1987^23^ | **2-point scale evaluated visually by laryngoscopy just prior to start of surgery**  None: No blood  Epistaxis-Trace: Visible blood only enough to coat inside of suction catheter or  Epistaxis-Measureable: Enough blood present to be suctioned into trap* | | |
|  |  |  |  |

*Five cases had measurable amounts of blood averaging at 2 ml and 19 cases had traces of blood. For this reason, the authors chose to combine the categories ‘Trace’ and ‘Measurable’ to the category ‘Epistaxis’ and compared this to the occurrences of ‘No blood’.
